# Polygenic Scores and Networks of Psychopathology Symptoms

**DOI:** 10.1001/jamapsychiatry.2024.1403

**Published:** 2024-06-12

**Authors:** Giulia G. Piazza, Andrea G. Allegrini, Thalia C. Eley, Sacha Epskamp, Eiko Fried, Adela-Maria Isvoranu, Jonathan P. Roiser, Jean-Baptiste Pingault

**Affiliations:** 1 Department of Clinical, Educational and Health Psychology, University College London, London, United Kingdom; 2 Institute of Cognitive Neuroscience, University College London, London, United Kingdom; 3 Department of Psychology, National University of Singapore, Singapore; 4 Social Genetic and Developmental Psychiatry, King’s College London, London, United Kingdom; 5 Department of Clinical Psychology, Leiden University, Leiden, the Netherlands

## Abstract

**Importance:**

Studies on polygenic risk for psychiatric traits commonly use a disorder-level approach to phenotyping, implicitly considering disorders as homogeneous constructs; however, symptom heterogeneity is ubiquitous, with many possible combinations of symptoms falling under the same disorder umbrella. Focusing on individual symptoms may shed light on the role of polygenic risk in psychopathology.

**Objective:**

To determine whether polygenic scores are associated with all symptoms of psychiatric disorders or with a subset of indicators and whether polygenic scores are associated with comorbid phenotypes via specific sets of relevant symptoms.

**Design, Setting, and Participants:**

Data from 2 population-based cohort studies were used in this cross-sectional study. Data from children in the Avon Longitudinal Study of Parents and Children (ALSPAC) were included in the primary analysis, and data from children in the Twins Early Development Study (TEDS) were included in confirmatory analyses. Data analysis was conducted from October 2021 to January 2024. Pregnant women based in the Southwest of England due to deliver in 1991 to 1992 were recruited in ALSPAC. Twins born in 1994 to 1996 were recruited in TEDS from population-based records. Participants with available genetic data and whose mothers completed the Short Mood and Feelings Questionnaire and the Strength and Difficulties Questionnaire when children were 11 years of age were included.

**Main Outcomes and Measures:**

Psychopathology relevant symptoms, such as hyperactivity, prosociality, depression, anxiety, and peer and conduct problems at age 11 years. Psychological networks were constructed including individual symptoms and polygenic scores for depression, anxiety, attention-deficit/hyperactivity disorder (ADHD), body mass index (BMI), and educational attainment in ALSPAC. Following a preregistered confirmatory analysis, network models were cross-validated in TEDS.

**Results:**

Included were 5521 participants from ALSPAC (mean [SD] age, 11.8 [0.14] years; 2777 [50.3%] female) and 4625 participants from TEDS (mean [SD] age, 11.27 [0.69] years; 2460 [53.2%] female). Polygenic scores were preferentially associated with restricted subsets of core symptoms and indirectly associated with other, more distal symptoms of psychopathology (network edges ranged between *r* = −0.074 and *r* = 0.073). Psychiatric polygenic scores were associated with specific cross-disorder symptoms, and nonpsychiatric polygenic scores were associated with a variety of indicators across disorders, suggesting a potential contribution of nonpsychiatric traits to comorbidity. For example, the polygenic score for ADHD was associated with a core ADHD symptom, being easily distracted (*r* = 0.07), and the polygenic score for BMI was associated with symptoms across disorders, including being bullied (*r* = 0.053) and not thinking things out (*r* = 0.041).

**Conclusions and Relevance:**

Genetic associations observed at the disorder level may hide symptom-level heterogeneity. A symptom-level approach may enable a better understanding of the role of polygenic risk in shaping psychopathology and comorbidity.

## Key Points

**Question:**

Which individual symptoms of psychopathology are associated with genetic risk?

**Findings:**

This cross-sectional study including a primary sample of 5521 individuals combined psychological network and polygenic score approaches and found polygenic scores for psychopathology-related traits were primarily associated with a restricted number of trait-relevant and cross-trait symptoms. Results were replicated in an independent sample of 4625 individuals following preregistered analyses.

**Meaning:**

A shift from thinking of psychopathology at the disorder level to thinking about individual transdiagnostic symptoms may be beneficial to uncover novel insights in the development and comorbidity of psychopathology; symptom-level analyses may be valuable in unraveling the complex (genetic) etiology of psychiatric conditions and avoiding pitfalls resulting from disorder heterogeneity.

## Teaser

This cross-sectional study evaluates associations between polygenic scores with psychiatric disorder symptoms and relevant comorbid phenotypes.

## Introduction

Genetic studies have consistently shown that many genetic variants, each exerting a small effect, are involved in complex human traits, and together contribute to the likelihood of developing psychiatric disorders.^1^ This polygenicity can be leveraged to compute polygenic scores (PGSs), weighted sums of risk variants carried by an individual.^2,3^ PGSs are a useful research tool indexing the genetic propensity to develop a particular psychiatric disorder, and have become instrumental in investigating the association between polygenic risk and psychiatric traits.

Findings based on PGSs partly depend on the operationalization of heterogeneous phenotypes. Notably, psychiatric disorders include a broad variety of symptoms, which, in combination, lead to numerous clinical presentations. This heterogeneity in psychiatric symptoms may bias genetic findings.^4^ In fact, evidence shows that symptoms have different heritability estimates, with some genetic effects specific to individual symptoms.^5,6,7^ Similarly, symptoms are differentially impacted by environmental risk factors and treatment, and contribute differently to relapse risk.^8,9,10^ In addition, some frequently comorbid disorders share a number of symptoms. For example, depression and anxiety frequently co-occur, and both feature insomnia, concentration problems, and fatigue.^11^ Findings on the shared genetic liability between comorbid disorders may therefore partly reflect a shared liability to transdiagnostic disorder features, such as endophenotypes or shared symptoms.

Therefore, analyzing unidimensional phenotypes, such as symptoms, can be more informative to uncover associations between biology and psychopathology^12^ by better capturing the heterogeneity of psychiatric traits.^13^ Psychological network modeling is a recently developed statistical framework used to explore associations between individual symptoms.^14^ Modeling observed variables as nodes (eg, individual items on psychological scales), and their statistical associations as edges (eg, partial correlations), networks allow for the visualization of reciprocal dependencies between symptoms, as well as exploratory and confirmatory analyses.^15^ By focusing on a more granular, symptom-based phenotype, incorporating PGSs in psychopathology networks can show whether PGSs are broadly associated with all facets of a trait or specifically with a restricted set of symptoms, and whether PGSs are associated with comorbid disorders via individual symptoms.

Here, we aimed to investigate how polygenic risk of psychopathology-related traits is associated with individual symptoms of childhood psychopathology. First, we examined the network structure of childhood behavioral and emotional symptoms, in combination with PGSs for depression, anxiety, and attention-deficit/hyperactivity disorder (ADHD), as well as body mass index (BMI) and educational attainment. Second, we tested how well our initial exploratory findings replicated in an independent sample with a preregistered confirmatory network analysis.

## Methods

### Sample

The Avon Longitudinal Study of Parents and Children (ALSPAC) is a large birth cohort study based in the Southwest of England that includes data on mothers, fathers, and children.^16,17^ Pregnant women residing in Avon and expected to deliver between 1991 and 1992 were recruited in the core sample (n = 14 541), followed by additional recruitment waves adding 906 pregnancies (14 901 children alive at age 1 year). Ethical approval for the study was obtained from the ALSPAC Ethics and Law Committee and the local research ethics committees. Written informed consent was obtained following the recommendations of the ALSPAC Ethics and Law Committee. The study website contains details of data that are available through a fully searchable data dictionary (https://www.bristol.ac.uk/alspac/researchers/our-data/).

For primary analyses, a sample of children with available genome-wide data was selected (n = 8365). Genotyping, imputation, and quality control steps for ALSPAC data are detailed in eMethods in Supplement 1. Questionnaires were sent out when children were 11 years old (n = 5521 from ALSPAC).

For replication analyses, a sample was selected from the Twins Early Development Study (TEDS), a large UK-based longitudinal study of families of twins born between 1994 and 1996 (n = 13 732).^18^ Identical selection steps were followed to match ALSPAC (n = 4625 from TEDS). Information on TEDS quality control is detailed by Selzam and colleagues.^19^

In both cohorts, only genotyped participants whose mothers responded to at least 75% of questionnaire items were included in the final analytical sample, retaining 5521 children from ALSPAC and 4625 from TEDS. Among these included individuals, we imputed remaining missing items using multiple imputation by predictive mean matching via the mice package version 3.14.0 in R (R Foundation). Of the maximum possible number of item data points (number of items × number of individuals), we imputed 0.73% of data points that were missing in ALSPAC and 0.1% in TEDS.

### Measures

Mother-rated reports of the Short Mood and Feelings Questionnaire (SMFQ, 13 items) and the Strength and Difficulties Questionnaire (SDQ, 25 items) were available in both ALSPAC and TEDS and were selected.^20,21^ Both are reliable and valid measures of, respectively, depression symptoms and social and emotional well-being, rated on a 3-point scale, 0 (not true), 1 (sometimes), or 2 (true). The SDQ is divided into 5 subscales: emotional problems, peer problems, hyperactivity, conduct problems, and prosociality. Following scoring guidelines, 5 SDQ items were reverse coded (items 7, 11, 14, 21, and 25). eTable 1 in Supplement 2 contains mean values and endorsement rates of SDQ and SMFQ (hereafter referred to as scale items). Items 1 and 4 of the SMFQ (miserable/unhappy and restless) were not present in TEDS and were therefore excluded in ALSPAC to match datasets, leaving 11 items of the SMFQ in the analysis.

### PGS Calculation

PGS for depression (based on genome-wide association study [GWAS] summary statistics^22^), anxiety,^23^ ADHD,^24^ BMI,^25^ and educational attainment^26^ were calculated using LDPred2^27^ in both cohorts. To ensure no overlap between target and base data, we selected summary statistics from large GWASs that did not include ALSPAC or TEDS in their samples. PGSs were generated by using the LDPred2-auto option with default parameters (using the R package bigsnpr version 1.10.8),^28^ limited to HapMap3 variants^29^ and using target data as reference linkage disequilibrium panels. Recommended quality control steps on GWAS summary statistics were performed prior to generating the scores^30^ (eMethods in Supplement 1).

### Covariates

To adjust for the effects of covariates on symptoms, age- and sex-regressed standardized residuals for each symptom were obtained from linear regressions and used as input data for networks in both cohorts. Scale items were adjusted for child age (around 11 years old) and sex. PGSs were adjusted for the first 10 genetic principal components, child age, sex, and genotyping chip and batch.

### Exploratory Network Estimation

Five cross-sectional networks with scale items and an individual PGS were estimated in ALSPAC (either depression, anxiety, ADHD, BMI, or educational attainment). Additional networks with all PGSs plus scale items and scale items only are available in the eResults and eFigure 3 in Supplement 1.

Unregularized model search was used for network estimation via the R package qgraph (version 1.9.2) and its ggmModSelect function,^31^ shown to perform optimally in large samples (N > 5000) compared to other network estimation techniques^32^ (eMethods in Supplement 1).

The resulting networks were visualized using the Fruchterman-Reingold algorithm.^33^ The accuracy of network parameters was investigated with the R package bootnet (version 1.5). One thousand nonparametric bootstraps were calculated for all network edge weights. Network weights matrices are reported in eTables 7-13 in Supplement 2. Additionally, we report covariate-adjusted correlations between PGSs and scale items (ie, correlations between each PGS and each scale item, only adjusted for covariates but not adjusted for all other associations between nodes, in contrast with network analyses) in eTable 14 in Supplement 2.

### Confirmatory Network Estimation

**Figure 1.**
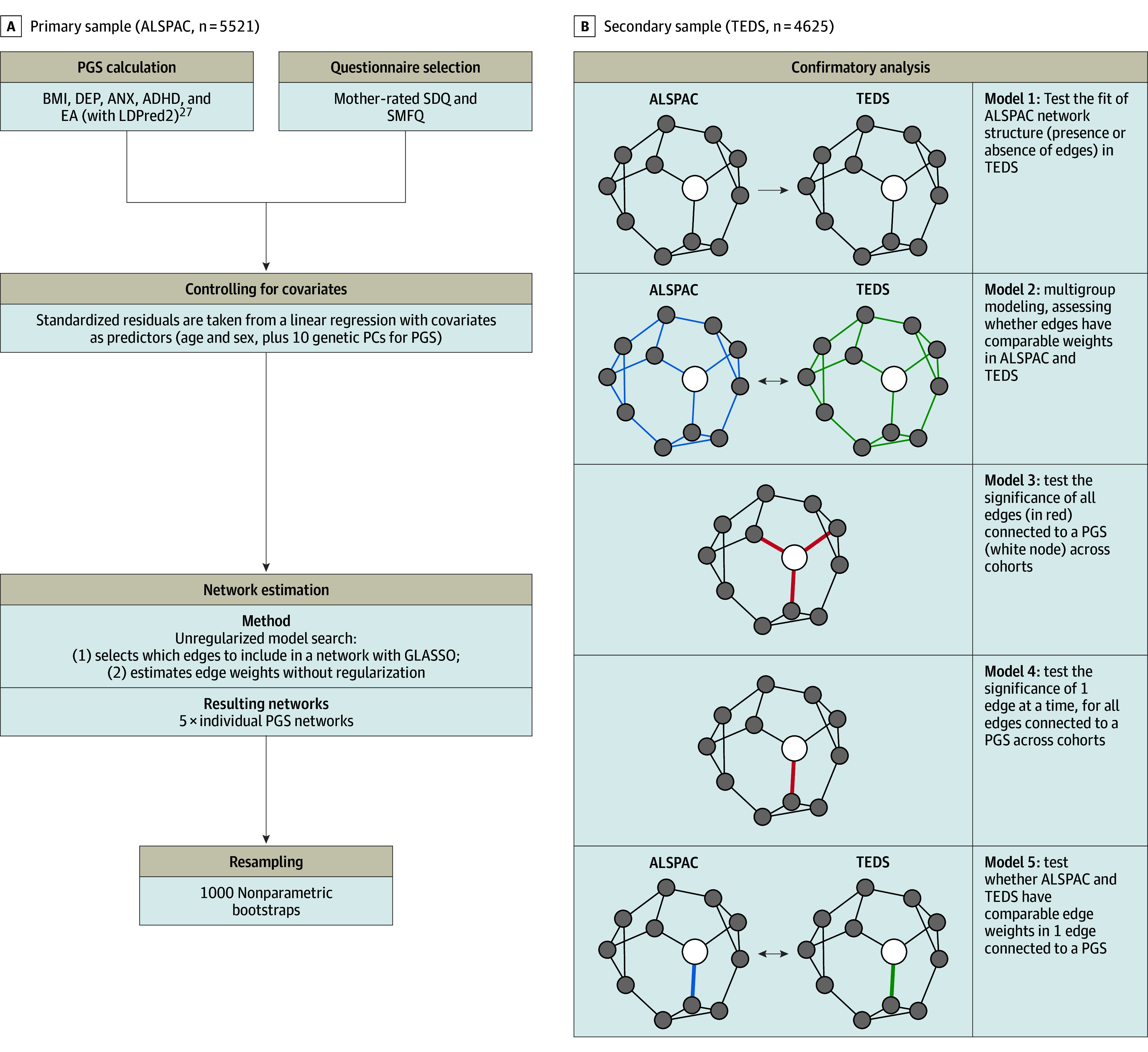
Analysis Flow of the Study, Including Network Analysis in the Avon Longitudinal Study of Parents and Children (ALSPAC) and Replication in the Twin Early Development Study (TEDS) ADHD indicates attention-deficit/hyperactivity disorder; ANX, anxiety; BMI, body mass index; DEP, depression; EA, educational attainment; GLASSO, graphical least absolute shrinkage and selection operator; PC, principal component; PGS, polygenic score; SDQ, Strength and Difficulties Questionnaire; SMFQ, Short Mood and Feelings Questionnaire.

We conducted a preregistered confirmatory analysis (https://osf.io/7y2g8) using the R package psychonetrics (version 0.10) (Figure 1).^14^ First, we tested whether the pattern of presence or absence of associations between items (network structure) was replicated in the secondary sample (model 1). Second, we tested whether the estimates of these associations (network edges) were comparable across samples (model 2). Third, we repeated these steps focusing particularly on associations between PGSs and symptoms (models 3-5).

Specifically, in model 1, we assessed how well network structures derived in the primary sample fit in our secondary sample using standard fit indices (root mean square error of approximation and comparative fit index). In model 2, in a combined dataset, we evaluated the fit of a model with equality constraints on network edges across cohorts, that is, a model in which all ALSPAC and TEDS edges were set to be equal. For example, we extracted the structure of the network with the ADHD PGS derived in ALSPAC and, in model 1, we tested the fit of this structure in TEDS. In model 2, we set all edges in the ADHD PGS network to have equal weights in ALSPAC and TEDS and evaluated model fit.

In model 3, we tested the overall significance of all edges connecting to the PGS node in a combined dataset. First, we estimated a model where all edges connecting the PGS were set to zero (model 3). For example, if the ADHD PGS was connected to the easily distracted and child cheats items in the primary results, both edges were set to zero. Second, we compared this to the original model, where these edges were retained as nonzero. In model 4, these steps were repeated on each edge connecting to PGSs. For example, we set the edge connecting the ADHD PGS to the easily distracted item to zero and compared this to the original model, which included the nonzero edge. Lastly, in model 5, individual edges connecting to PGSs were free to vary between cohorts. For example, the edge connecting the ADHD PGS to the easily distracted item was allowed to freely vary between ALSPAC and TEDS. We compared this to a model where this edge was set to be equal.* P* values were adjusted for multiple comparisons with false discovery rate correction using the Benjamini-Hochberg method (α = .05) and the R package stats (version 4.2.0) in model 4 (34 tests) and model 5 (35 tests).^34^

### Statistical Analyses

All analyses were carried out with R version 4.2.0 (R Foundation), outlined in Figure 1, and reported following the Strengthening the Reporting of Observational Studies in Epidemiology (STROBE) reporting guideline (eTable 15 in Supplement 2). Example code is available on GitHub.

## Results

### Exploratory Analyses

**Figure 2.**
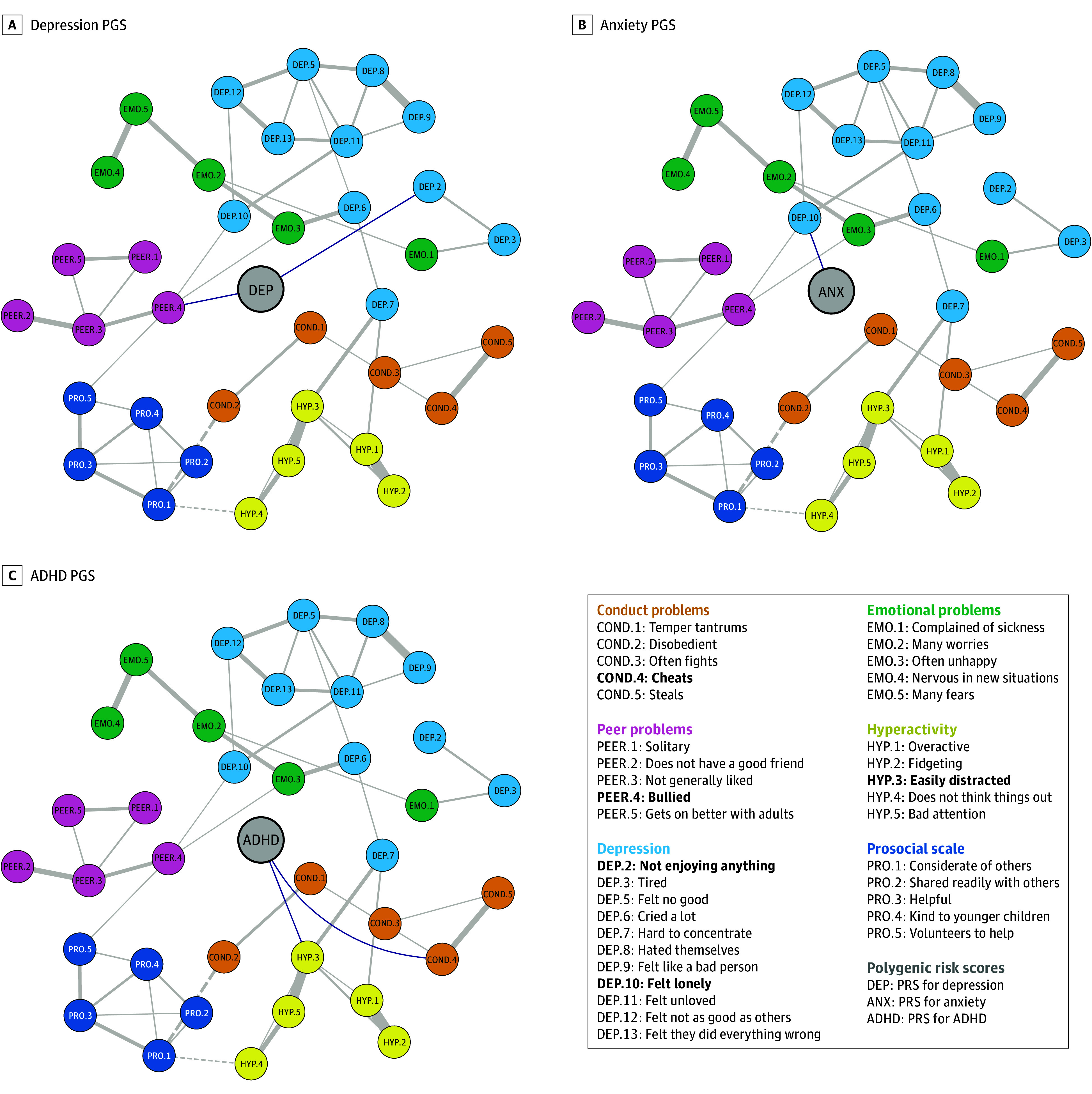
Networks of Psychiatric Polygenic Scores (PGSs) and Psychopathology Symptoms Plots of networks with depression PGS, anxiety PGS, attention-deficit/hyperactivity disorder (ADHD) PGS. Partial correlations between scale items are drawn in the plot when |*r*| > 0.1 for clarity (ie, the threshold for qgraph visualization of edges connecting scale items is 0.1). All partial correlations between PGS nodes and scale items are drawn (ie, qgraph visualization threshold is 0 for edges connecting PGS). Blue edges connecting PGSs indicate positive associations. All edges connecting scale items are solid gray when positive and dotted gray when negative. Bold items in the legend indicate nodes connected to a PGS. PGSs are in the center of each graph, and all other nodes are positioned according to an average layout obtained with the Fruchterman-Reingold algorithm. eFigure 1 in Supplement 1 includes all networks without thresholds and common layout. ANX indicates anxiety; COND, conduct problems; DEP, depression; EMO, emotional problems; HYP, hyperactivity problems; PEER, peer problems; PRO, prosocial scale.

The exploratory population included 5521 participants from ALSPAC (mean [SD] age, 11.8 [0.14] years; 2777 [50.3%] female). PGSs were preferentially associated with specific items of their corresponding traits. For example, the ADHD PGS (Figure 2C) was only associated with 1 hyperactivity item: easily distracted (*r* = 0.07), and the depression PGS (Figure 2A) was associated with the depression symptom not enjoying anything (*r* = 0.04).

Additionally, psychiatric PGSs were not associated only with trait-concordant items but showed cross-trait associations. For example, in addition to its within-trait associations, the ADHD PGS was also associated with the child cheats item (*r* = 0.05) in the conduct problems subscale, and the depression PGS was also associated with being bullied (*r* = 0.06) in the peer problems subscale. Similarly, the anxiety PGS was associated with depression node feeling lonely (*r* = 0.04) (Figure 2B). Moreover, PGSs were associated with a broader set of items based on covariate-adjusted correlations (ie, adjusted for covariates, but not adjusted for all associations between nodes as in network analyses) (eTable 14 in Supplement 2).

**Figure 3.**
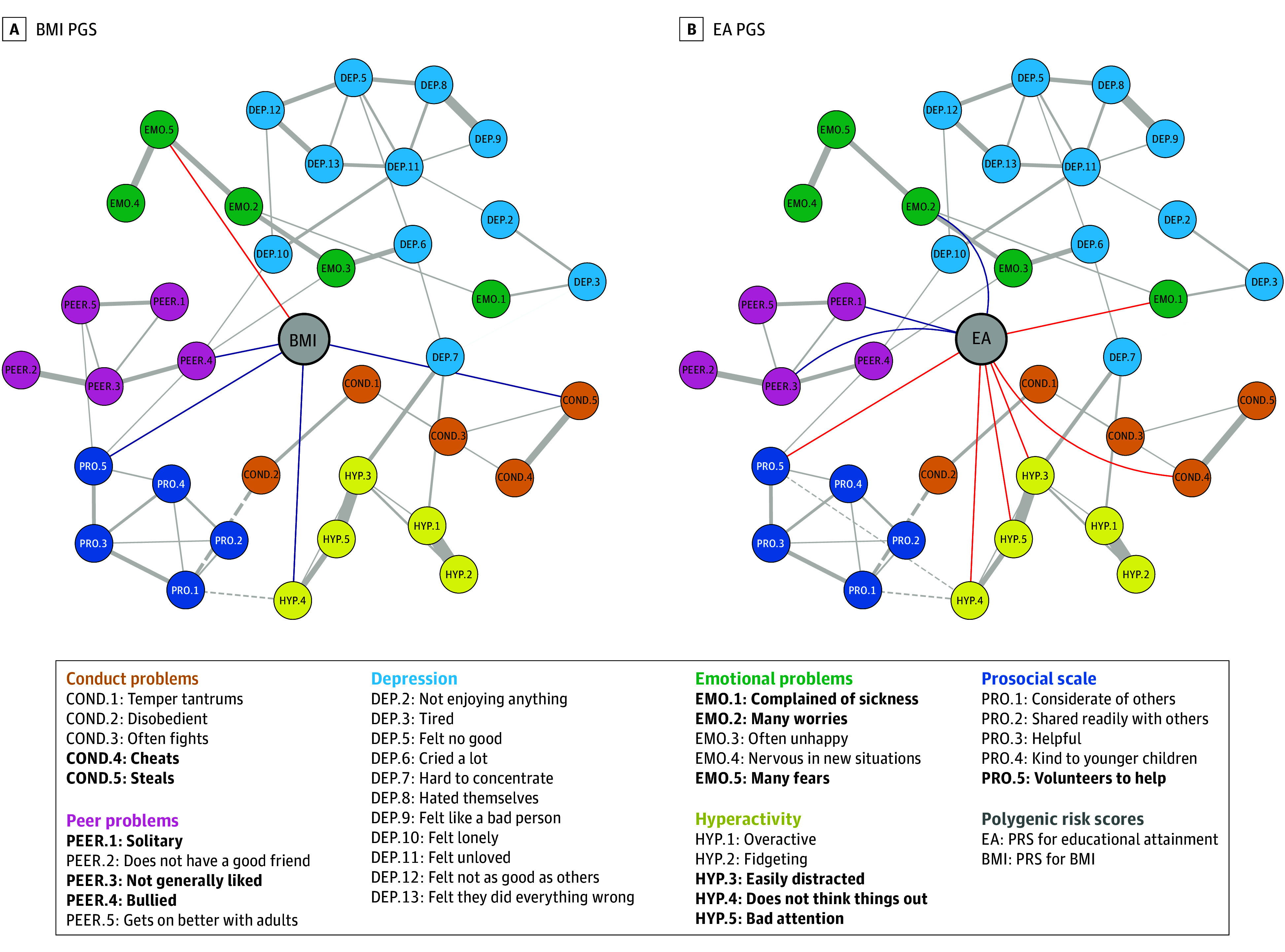
Networks of Nonpsychiatric Polygenic Scores (PGSs) and Psychopathology Symptoms Plots of networks with body mass index (BMI) PGS and educational attainment (EA) PGS. Partial correlations between scale items are drawn in the plot when |r| > 0.1 for clarity, and all partial correlations between PGS nodes and scale items are drawn. All edges connecting PGS are blue when positive and red when negative. All edges connecting scale items are solid gray when positive and dotted gray when negative. Bold items in the legend indicate nodes connected to a PGS. PGSs are in the center of each graph and all other nodes are positioned according to an average layout obtained with the Fruchterman-Reingold algorithm. COND indicates conduct problems; DEP, depression; EMO, emotional problems; HYP, hyperactivity problems; PEER, peer problems; PRO, prosocial scale.

Lastly, nonpsychiatric traits were associated with symptoms across disorders. The BMI PGS (Figure 3A) was positively associated with conduct, peer, prosociality, and hyperactivity problems and negatively associated with emotional issues. The educational attainment PGS was negatively associated with items belonging to most subscales, as well as most hyperactivity problems (Figure 3B). Nonparametric bootstraps showed edges were estimated accurately, as sample values were comparable to bootstrap mean edge weights (eFigure 2 in Supplement 1).

### Confirmatory Analyses

The confirmatory population included 4625 participants from TEDS (mean [SD] age, 11.27 [0.69] years; 2460 [53.2%] female). Overall, networks replicated well across datasets. Models 1 and 2 indicated network models were successfully replicated in the secondary sample. All network structures derived in ALSPAC showed good model fit in TEDS based on standard fit indices in model 1 (CFI > 0.95; RMSEA < 0.05) (Table 1). Similarly, when setting equality constraints between ALSPAC and TEDS edges (model 2), model fit was good across all networks (eTable 3 in Supplement 2). Although standard fit indices were comparatively better when edges were not constrained to be equal across samples, indices accounting for model complexity (eg, the bayesian information criterion) consistently favored models with constrained edges.

**Table 1.** Model Fit Indices From Model 1, Testing the Model Fit of the Avon Longitudinal Study of Parents and Children (ALSPAC) Networks in the Twin Early Development Study (TEDS)

Fit index	Network				
	ADHD PGS	Depression PGS	Anxiety PGS	EA PGS	BMI PGS
CFI^a^	0.977	0.979	0.978	0.978	0.977
RMSEA^a^	0.021	0.020	0.021	0.021	0.021

Abbreviations: ADHD, attention-deficit/hyperactivity disorder; BMI, body mass index; CFI, comparative fit index; EA, educational attainment; PGS, polygenic score; RMSEA, root mean square error of approximation.

^a^ CFI above 0.95 and RMSEA below 0.05 were considered indicators of good model fit and of successful replication of ALSPAC networks in TEDS.

Edges connecting PGSs were statistically significant in all networks, as models including these edges (models 3 and 4) fit better than models that excluded them (eTables 4-5 in Supplement 2). In addition, results from model 5 show that PGS had similar associations with items across cohorts. Models constraining PGS edges to be equal in ALSPAC and TEDS were preferred to models that lifted these equality constraints, except the edge between the educational attainment PGS and child cheats item. However, this difference did not survive corrections for multiple comparisons.

## Discussion

This cross-sectional study examined the associations between childhood psychopathology symptoms and PGSs for psychiatric disorders and relevant traits using a network approach. We found that psychiatric PGSs were associated with a core subset of indicators of their corresponding traits and that PGSs were not only associated with symptoms of their respective trait but showed direct cross-trait associations. These findings were replicated in an independent sample and, as discussed below, suggest that the association between psychiatric and nonpsychiatric polygenic risk and psychopathology traits may be mediated by specific factors or other symptoms.

### Trait-Relevant Associations Between PGSs and Symptoms

PGSs were associated with a selection of items measuring their corresponding trait. For example, the ADHD PGS was only positively associated with 1 item in the hyperactivity subscale (easily distracted). This result suggests the association between ADHD and the polygenic risk for ADHD might be preferentially explained by the association with cognitive-attentional elements of the disorder. Similarly, the depression PGS was positively associated with anhedonia (not enjoying anything), suggesting the polygenic risk for depression might primarily influence prominent features of the disorder associated with the greatest impairment.^35^ When associations between items and PGSs were not adjusted for all associations between network nodes (ie, in covariate-adjusted correlations; eTable 14 in Supplement 2), PGSs were associated with a broader set of items than those identified by network analysis.

Taken together, these results suggest that associations between PGSs and psychiatric traits might be preferentially explained by the association with core symptoms, rather than reflect uniform associations with all symptoms as commonly implied by disorder-level analyses. These core symptoms may be key mediators in the relationships between PGSs and other, more distal symptoms of psychopathology.

### Cross-Trait Associations Between PGSs and Symptoms

PGSs for psychiatric disorders were also found to be associated with items that did not directly measure PGS-concordant phenotypes. Notably, the anxiety PGS was associated with depression symptom of feeling lonely. This may indicate that a shared genetic influence on individual symptoms of depression and anxiety contributes to their frequent co-occurrence.

Similarly, the educational attainment PGS was negatively associated with individual hyperactivity items. Previous evidence suggests higher educational attainment PGS predict lower ADHD symptoms and better inhibitory control.^36^ Indeed, our networks showed the educational attainment PGS was negatively associated with cheating, having poor attention, and being easily distracted and impulsive and positively associated with internalizing and peer problems, such as being solitary, having many worries, not being liked, and not volunteering to help others. This may suggest that childhood educational attainment is a reflection of social and cognitive processes that also play a part in most internalizing and externalizing disorders.

Furthermore, the BMI and depression PGSs were associated with peer problems, specifically with being bullied. In turn, being bullied was positively associated with being lonely and often unhappy, suggesting that being bullied may mediate the association between these PGSs and depression symptoms. This is also consistent with recent evidence showing the genetic predisposition to higher BMI, depression, and ADHD is associated with bullying victimization in children.^37^ Pre-existing vulnerability to mental illness might lead to exposure to bullying in childhood, which in turn exacerbates emotional difficulties in adolescence,^38^ hyperactivity and impulsivity, inattention, and conduct problems.^39^ This represents a pattern of evocative gene-environment correlation: children who are predisposed to developing a high BMI might, in some contexts, evoke particular reactions in their environment, such as bullying.^40^ Unfavorable environments, in turn, affect mental and physical health. This can have cascading effects, as stress in early life mediates the association between the genetic predisposition to high BMI and later depression.^41^

In sum, adopting a network approach to phenotyping can suggest potential pathways to developing psychiatric traits by highlighting indirect paths from polygenic risk to later psychopathology via intermediate phenotypes. Taking a dimensional view of psychopathology, we investigated the extent to which common genetic variation in the population (indexed by PGSs) is associated with individual differences in symptoms. Findings should be replicated in high-risk or clinical cohorts.

### Limitations

A few limitations of this study merit comment. First, the partial correlations evidenced in our study cannot be assumed to reflect causal mechanisms.

Second, results derived from our discovery cohort (ALSPAC) may be affected by overfitting, which could affect results in the combined sample of both cohorts (models 3 and 4). As such, edges between PGSs and scale items derived in the confirmatory sample are the most conservative estimates (Table 2). Models investigating differences in edges between cohorts (model 5) were implemented to minimize this issue. In fact, we did not observe any systematic deflation of estimates in the second cohort, reducing the likelihood of inflated estimates in the discovery cohort.

**Table 2.** Weights (Partial Correlations) of the Edges of Interest in Polygenic Score (PGS) Networks

Network and items	Edge	Weight^a^		
		ALSPAC	TEDS	Constrained model
EA PGS network				
Cheats^b^	COND.4–EA^b^	−0.049	−0.098	−0.072
Complained of sickness	EMO.1–EA	−0.044	−0.014	−0.031
Many worries	EMO.2–EA	0.040	0.053	0.046
Easily distracted	HYP.3–EA	−0.062	−0.044	−0.054
Does not think things out	HYP.4–EA	−0.052	−0.028	−0.040
Bad attention	HYP.5–EA	−0.048	−0.069	−0.058
Solitary	PEER.1–EA	0.037	0.010	0.025
Not generally liked	PEER.3–EA	0.036	0.027	0.033
Volunteers to help	PRO.5–EA	−0.078	−0.069	−0.074
BMI PGS network				
Steals	COND.5–BMI	0.048	0.039	0.044
Many fears	EMO.5–BMI	−0.039	−0.011	−0.026
Does not think things out	HYP.4–BMI	0.043	0.038	0.041
Bullied	PEER.4–BMI	0.051	0.054	0.053
Volunteers to help	PRO.5–BMI	0.074	0.073	0.073
ADHD PGS network				
Cheats	COND.4–ADHD	0.048	0.040	0.044
Easily distracted	HYP.3–ADHD	0.070	0.069	0.070
Depression PGS network				
Not enjoying anything	DEP.2–DEP	0.037	0.037	0.038
Bullied	PEER.4–DEP	0.055	0.036	0.047
Anxiety PGS network				
Felt lonely	DEP.10–ANX	0.040	0.014	0.028

Abbreviations: ADHD, attention-deficit/hyperactivity disorder; ALSPAC, the Avon Longitudinal Study of Parents and Children; BMI, body mass index; COND, conduct problems; COND.4, Cheats; COND.5, steals; DEP, depression; DEP.10, felt lonely; DEP.2, not enjoying anything; EA, educational attainment; EMO, emotional problems; EMO.1, complained of sickness; EMO.2, many worries; EMO.5, many fears; HYP, hyperactivity problems; HYP.3, easily distracted; HYP.4, does not think things out; HYP.5, bad attention; PEER, peer problems; PEER.1, solitary; PEER.3, not generally liked; PEER.4, bullied; PRO, prosocial scale; PRO.5, volunteers to help; TEDS, the Twin Early Development Study.

^a^ Weights were derived from primary analyses (ALSPAC weights), confirmatory model 1 (TEDS weights), and confirmatory model 2 (constrained model weights).

^b^ Significantly different weight estimates in TEDS and ALSPAC based on uncorrected *P* values in model 5. When correcting for multiple comparisons, the difference is nonsignificant. All other estimates are not significantly different in TEDS and ALSPAC based on both uncorrected and corrected *P* values.

Third, polygenic scoring is a proxy for individual genetic liability, and it does not capture the full heritability of a trait (single-nucleotide variant heritability) due to measurement error, meaning there are likely associations between genetic liabilities and symptoms that our analysis was not able to detect. The PGS calculated in this study vary in predictive power, in accordance with the GWAS they were derived from. This may explain some findings, such as the educational attainment PGS associating with more symptoms of ADHD than the ADHD PGS itself.

Additionally, ALSPAC and TEDS are affected by attrition.^16,17,18^ Therefore, replications of these findings in representative cohorts with high retention rates are warranted. Similarly, this analysis was limited to participants of European descent. As more diverse samples are being made available for genetic research, it will be important to verify whether our findings hold true in those samples. Replication studies would also benefit from using more normally distributed item data and more granular genetic data (eg, symptom-level GWAS, eDiscussion in Supplement 1).

## Conclusions

Modeling polygenic risk in networks of psychological variables showed previously unreported patterns of associations that replicated across samples. Associations between psychopathology-associated PGSs and childhood psychological difficulties suggest that PGSs are preferentially associated with specific trait-relevant and cross-trait symptoms. Introducing genetic data into psychological networks can provide new insights into the etiology of comorbidity as well as identify potential pathways to the development of psychiatric traits.

## Notes

Supplement 1. eMethods eResults eDiscussion eFigure 1. Networks of PGS and psychopathology symptoms without formatting to highlight PGS eFigure 2. Weights (partial correlations) of edges connecting PGS nodes in their respective networks eFigure 3. Plot of phenotypic network and all PGS network eReferences

Supplement 2. eTable 1. Items of the SDQ and SMFQ, with node abbreviations, endorsement frequencies, sample size (of genotyped individuals), mean and standard deviations eTable 2. Number of SNPs resulting from GWAS QC procedures and SNPs included in polygenic scores (PGS). eTable 3. Model fit indices for Model 2 in all networks and model comparisons between constraned and unconstrained models in all networks. eTable 4. Model 3 results: model comparisons between models with all PGS edges, and models without PGS edges in each network. eTable 5. Model 4 results: model comparisons between models with all PRS edges (original), and models without a single PRS edge in each network. eTable 6. Model 5 results: model comparisons between models where PRS edges are equal between ALSPAC and TEDS (original) and models where they are free to vary. eTable 7. BMI PGS network matrix eTable 8. Depression PGS network matrix eTable 9. EA PGS network matrix eTable 10. Anxiety PGS network matrix eTable 11. ADHD PGS network matrix eTable 12. All PGS network matrix eTable 13. Phenotypic network matrix eTable 14. Covariate-adjusted correlations between polygenic scores and scale items eTable 15. STROBE Statement—Checklist of items that should be included in reports of cohort studies

Supplement 3. Data sharing statement
