# Supplementary material for: Polygenic Scores and Networks of Psychopathology Symptoms
Source: JAMA Psychiatry. 2024 Jun 12;81(9):902–10. doi: 10.1001/jamapsychiatry.2024.1403 (PMC11170456; doi:10.1001/jamapsychiatry.2024.1403)
Supplement: Supplement 1. — eMethods eResults eDiscussion eFigure 1. Networks of PGS and psychopathology symptoms without formatting to highlight PGS eFigure 2. Weights (partial correlations) of edges connecting PGS nodes in their respective networks eFigure 3. Plot of phenotypic network and all PGS network eReferences [file jamapsychiatry-e241403-s001.pdf]

Supplemental Online Content

Piazza GG, Allegrini AG, Eley TC, et al. Polygenic scores and networks of psychopathology symptoms. *JAMA Psychiatry*. Published online June 12, 2024. doi:10.1001/jamapsychiatry.2024.1403

eMethods
eResults
eDiscussion
eFigure 1. Networks of PGS and psychopathology symptoms without formatting to highlight PGS
eFigure 2. Weights (partial correlations) of edges connecting PGS nodes in their respective networks
eFigure 3. Plot of phenotypic network and all PGS network
eReferences

This supplemental material has been provided by the authors to give readers additional information about their work.

© 2024 American Medical Association. All rights reserved.

eMethods

Quality control for PGS

We performed standard quality control procedures on GWAS summary statistics and target data.[1] When the information was provided by GWAS authors, SNPs with INFO scores below 0.8 and Minor Allele Frequency (MAF) below 0.01 were excluded, along with ambiguous and duplicate SNPs (see eTable 2). ALSPAC provides genotype information for children of European ancestry (as detailed here https://www.bristol.ac.uk/media-library/sites/alspac/migrated/documents/gwas-data-generation.pdf?u07022013) and TEDS provides genotype data for children of white ethnicity (as detailed here https://www.teds.ac.uk/datadictionary/exclusions.htm). Our analysis was therefore restricted to these participants.

In ALSPAC genetic data, we removed non-autosomal SNPs, as well as SNPs and individuals with high levels of missingness (more than 5% missing). Related individuals (10% or more alleles shared Identity By Descent), individuals with discordant sex information and heterozygosity rate more than 3 standard deviations from the mean were excluded. SNPs with a MAF < 0.01 and significantly deviating from Hardy-Weinberg Equilibrium ($p < 1e-7$) were excluded, leaving 4,886,821 SNPs. We calculated polygenic scores by filtering HapMap3 SNPs and computing a Linkage Disequilibrium (LD) reference from our data.[2] In TEDS, UK Biobank was used as LD reference.[3]

PGS calculation

PGS were calculate with LDPred2, a Bayesian method to derive polygenic scores using information on genetic architecture (SNP-heritability), on the fraction of causal variants (polygenicity) and on LD obtained from a reference panel.[4]

Target data (ALSPAC and TEDS) were used as reference LD panels in PGS calculations. PGS were generated by using the option 'LDPred2-auto'. To compute PGS, the recommended steps by the LDPred2 development team[2] were followed, and, accordingly, variants included were restricted to HapMap3 variants (https://www.sanger.ac.uk/resources/downloads/human/hapmap3.html). Example code is available on GitHub[5].

Network estimation

Unregularised model search was used for network estimation ('ggmModSelect' in *qgraph*[6]). The algorithm selects which edges to include in a network and estimates their weights. One hundred networks are initially estimated, ranging from very sparse (i.e., with few edges) to very dense, using the graphical least absolute shrinkage and selection operator (gLASSO). LASSO regularisation sets an upper bound to the total sum of parameters in the network, with the aim of minimising the number of spurious edges (for more details, see Epskamp et al.[6]). A set of edges to include is thus obtained for each of the 100 networks. Models are subsequently re-fit without regularisation to compute the weights of included edges. The network with the optimal model is chosen by minimising the Extended Bayesian Information Criterion (EBIC). In a final step, individual edges are progressively added or removed to further improve fit (stepwise estimation).

eResults

All PGS network

The inclusion of all PGS simultaneously in one network did not fundamentally change results (eFigure 3b). Exceptions were the edges connecting the BMI PGS to items '*Does not think things out*' (HYP.4) and '*Steals'* (COND.5), the anxiety PGS to item *'Feeling lonely'* (DEP.10), and the depression PGS to item '*Not enjoying anything'* (DEP.2). An additional edge between the PGS for depression and item '*Child has many worries*' (EMO.2) was observed. Results indicated that network structure and weights were successfully replicated. Network structures had good model fit in the secondary sample (model 1; $CFI \geq 0.98$, $RMSEA \leq 0.019$). Constraining edges to be equal between cohorts resulted in good model fit (model 2, eTable 3), and this was the best fitting and most parsimonious model according to the BIC (eTable 3).

Additionally, all associations between PGS and scale items were statistically significant and of similar magnitude in both cohorts. Models including edges connecting PGS (models 3 and 4) were preferred to those excluding them. All edges connecting PGS were of similar magnitude in TEDS and ALSPAC (model 5), with the exception of the edge between the PGS for EA and item '*Child cheats*' (COND.4). However, this difference did not survive corrections for multiple comparisons.

© 2024 American Medical Association. All rights reserved.

Phenotypic network

Behavioural and emotional symptoms of psychopathology were frequently positively correlated (eFigure 3a), not only within individual subscales (e.g., correlations among 'Peer problems' items), but also between scales (e.g., between 'Peer problems' and 'Depression' items). Overall, non-zero network edges varied in weight. The strongest positive partial correlation between phenotypic items was between node *'Overactive'* (HYP.1) and *'Fidgeting'* (HYP.2), r = 0.46, while the strongest negative partial correlation was between node *'Considerate of others'* (PRO.1) and *'Disobedient'* (COND.2), r = -0.17. Results indicated the phenotypic network model was successfully replicated in the secondary sample, with similar associations between scale items in both samples. The structure of this network showed good model fit when tested in the secondary sample (model 1, CFI ≥ 0.98, RMSEA ≤ 0.021), based on standard fit indices thresholds. Constraining edges to be equal across cohorts resulted in good model fit (model 2, eTable 3), and the model where edges were constrained to be equal between cohorts was the best fitting and most parsimonious according to the BIC.

eDiscussion

We note additional limitations of the study. PGS are themselves sum-scores, and therefore, not unlike sum scores on psychopathology scales, they might hide the complexity of the genetic architecture of disorders (e.g. interactions). Moreover, findings are dependent on PGS derived from disorder-level GWAS. Future efforts may benefit from considering symptom-level approaches to phenotyping, such as symptom-level GWAS and network modelling.

© 2024 American Medical Association. All rights reserved.

eFigures

eFigure 1 a-g: Networks of PGS and psychopathology symptoms without formatting to highlight PGS.

Plots for networks with the polygenic score for EA (a), BMI (b), anxiety (c), ADHD (d), depression (e), all polygenic scores (f) and phenotypic network (g). Positive correlations are in blue and negative in red. Please refer to eTable 1 for node abbreviations.

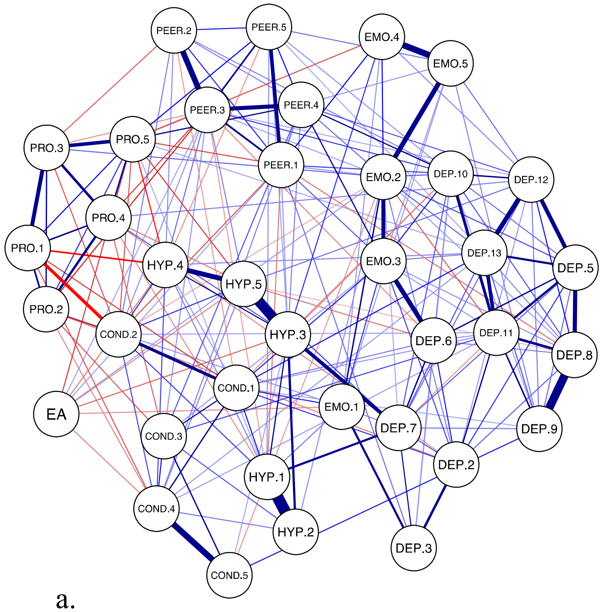

a.

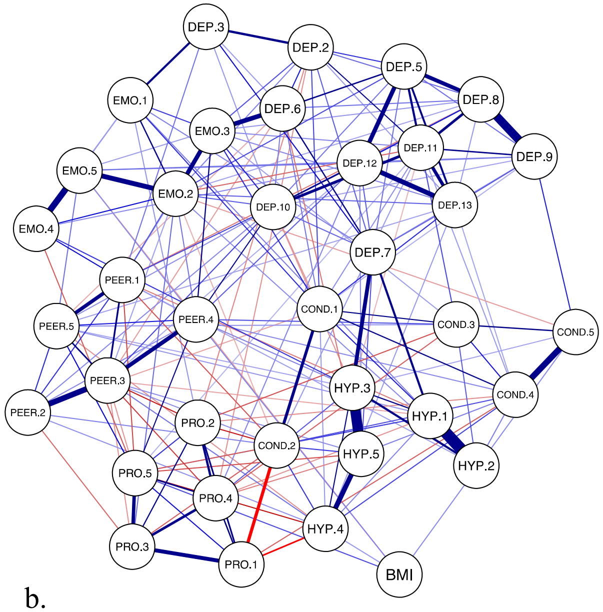

b.

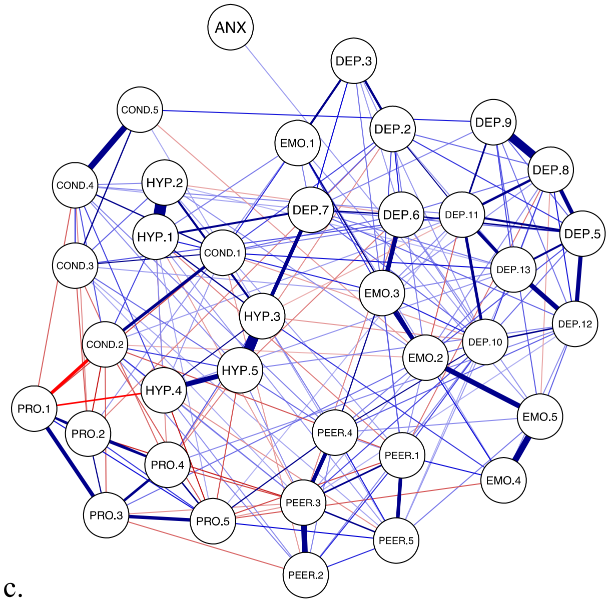

c.

© 2024 American Medical Association. All rights reserved.

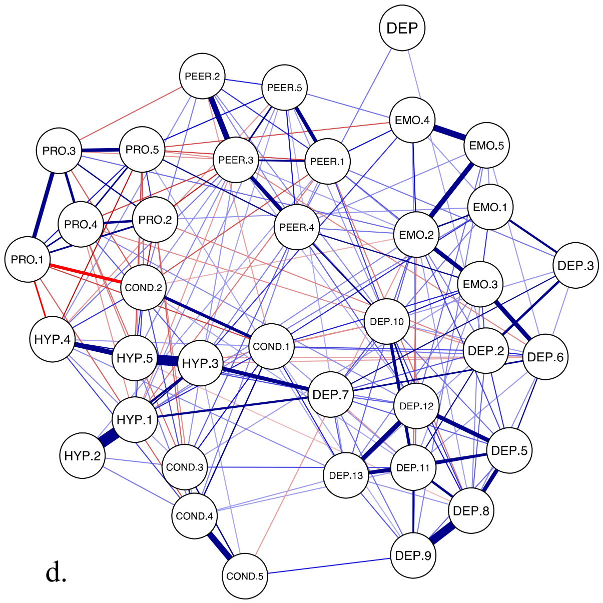
DEP
PEER.2
PEER.5
EMO.4
EMO.5
PRO.3
PRO.5
PEER.3
PEER.1
EMO.1
PRO.4
PRO.2
PEER.4
EMO.2
PRO.1
COND.2
EMO.3
DEP.3
DEP.10
HYP.4
HYP.5
HYP.3
COND.1
DEP.2
DEP.6
DEP.7
DEP.12
HYP.1
HYP.2
COND.3
DEP.13
DEP.11
DEP.5
COND.4
DEP.8
DEP.9
COND.5

d.

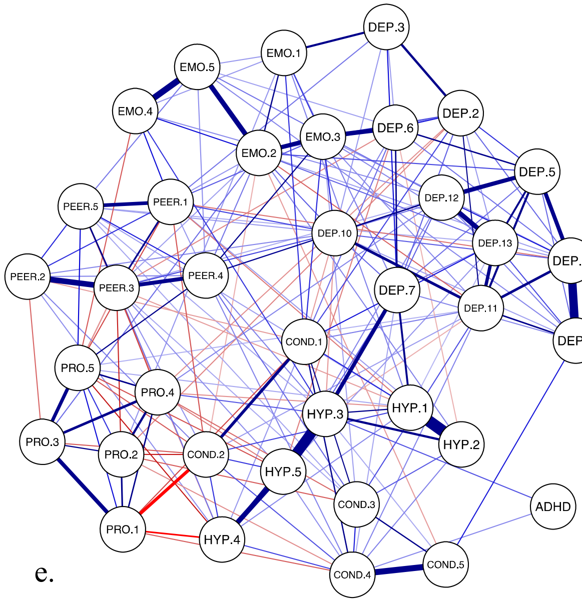
DEP.3
EMO.5
EMO.1
EMO.4
DEP.2
EMO.3
DEP.6
EMO.2
DEP.5
PEER.5
PEER.1
DEP.12
DEP.10
DEP.13
DEP.8
PEER.2
PEER.3
PEER.4
DEP.7
DEP.11
DEP.9
COND.1
PRO.5
PRO.4
HYP.1
HYP.3
PRO.3
PRO.2
COND.2
HYP.2
HYP.5
COND.3
ADHD
PRO.1
HYP.4
COND.4
COND.5

e.

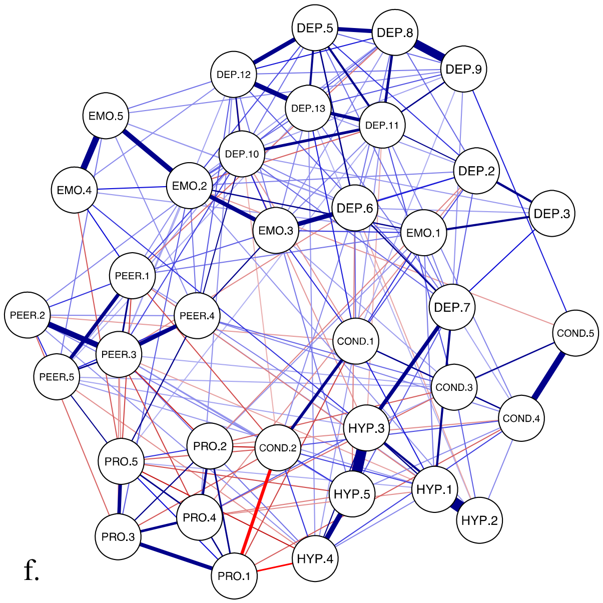
DEP.5
DEP.8
DEP.12
DEP.9
DEP.13
DEP.11
EMO.5
DEP.10
DEP.2
EMO.4
EMO.2
DEP.6
DEP.3
EMO.3
EMO.1
PEER.1
PEER.2
PEER.4
DEP.7
COND.1
COND.5
PEER.3
PEER.5
COND.3
COND.4
HYP.3
PRO.2
COND.2
PRO.5
HYP.5
HYP.1
PRO.4
HYP.2
PRO.3
HYP.4
PRO.1

f.

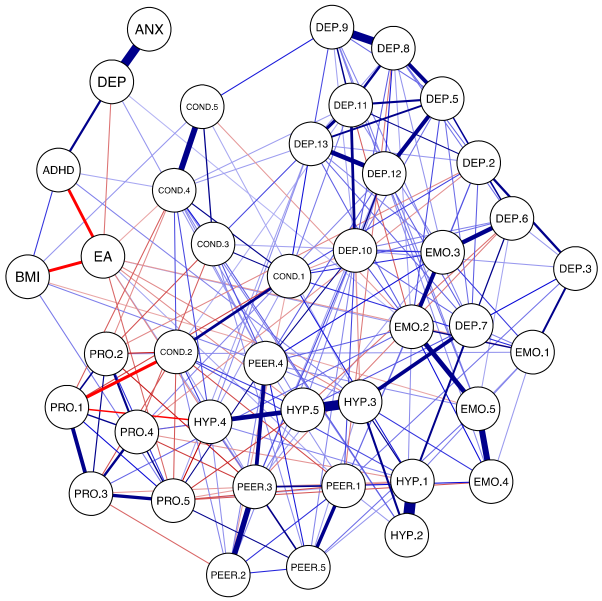
ANX
DEP.9
DEP.8
DEP
COND.5
DEP.11
DEP.5
DEP.13
DEP.12
DEP.2
ADHD
COND.4
DEP.6
COND.3
DEP.10
EMO.3
EA
BMI
COND.1
DEP.3
EMO.2
DEP.7
COND.2
PRO.2
PEER.4
EMO.1
PRO.1
HYP.5
HYP.3
EMO.5
PRO.4
HYP.4
PEER.3
PEER.1
HYP.1
EMO.4
PRO.3
PRO.5
HYP.2
PEER.2
PEER.5

g.

ed.

eFigure 2: **Weights (partial correlations) of edges connecting PGS nodes in their respective networks.**

Weights were derived from primary networks (ALSPAC edge weights, in blue). Mean bootstrap edge weights (in orange) and their quantile ranges (black lines) were derived from 1000 non-parametric bootstraps. Boxes on the right indicate the proportion of times edges were not included in bootstrap networks. Please refer to Table 2 for node abbreviations.

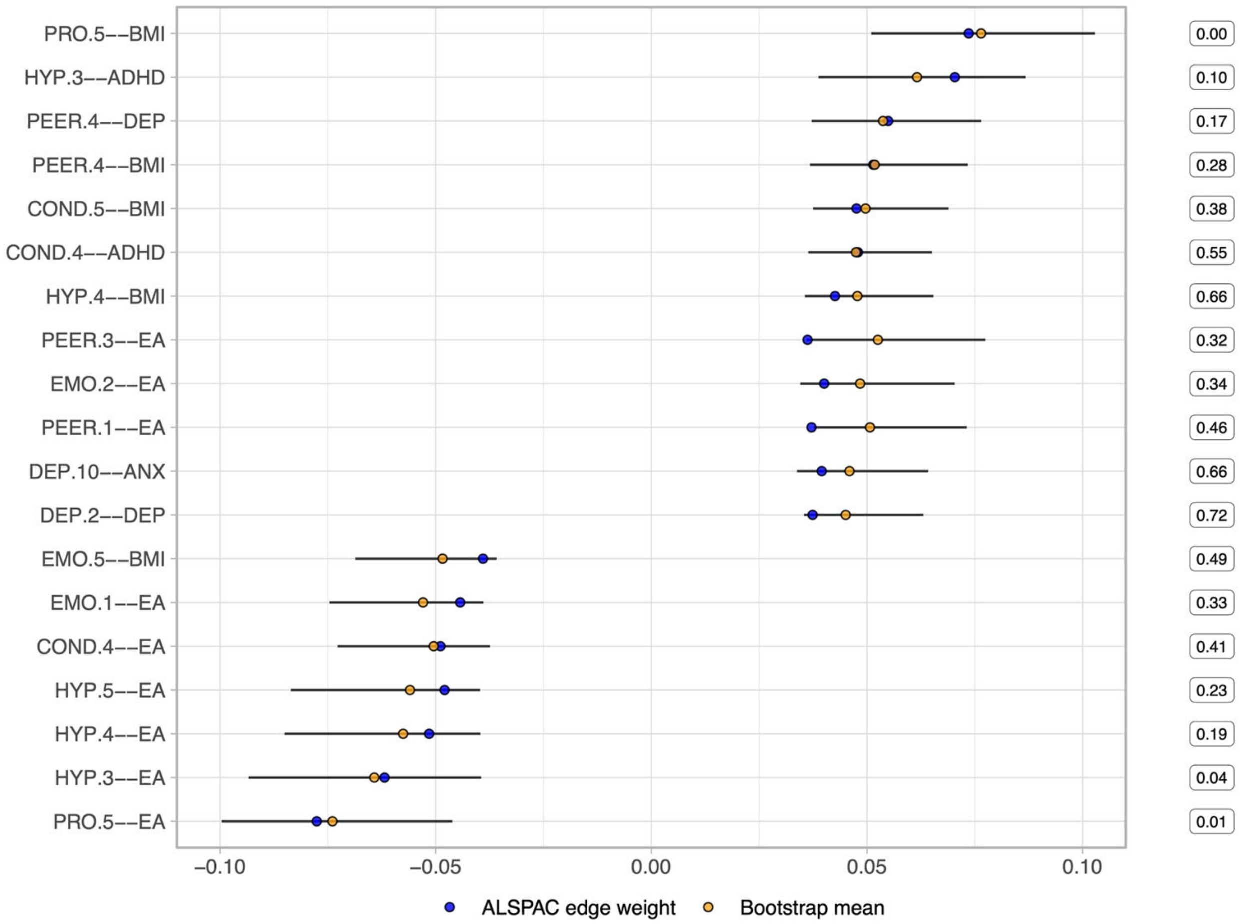

© 2024 American Medical Association. All rights reserved.

eFigure 3 a-b: Plot of phenotypic network (a) and all PGS network (b).

In (a), partial correlations between scale items are drawn when |r| > 0.1 for clarity (threshold for *qgraph* visualization = 0.1). All edges are blue when positive and red when negative. In (b), all partial correlations are drawn (*qgraph* visualization threshold = 0). Edges connecting scale items are solid grey when positive and dotted grey when negative. Bold items in the legend indicate nodes connected to a PGS.

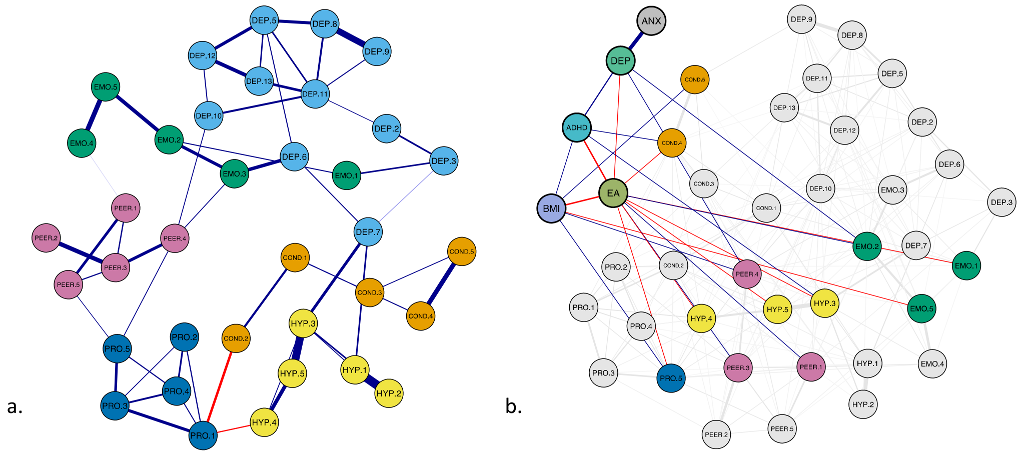

Conduct Problems
- COND.1: Temper tantrums
- COND.2: Disobedient
- COND.3: Often fights
- **COND.4: Cheats**
- **COND.5: Steals**

Peer Problems
- **PEER.1: Solitary**
- PEER.2: Does not have a good friend
- **PEER.3: Not generally liked**
- **PEER.4: Bullied**
- PEER.5: Gets on better with adults

Depression
- DEP.2: Not enjoying anything
- DEP.3: Tired
- DEP.5: Felt no good
- DEP.6: Cried a lot
- DEP.7: Hard to concentrate
- DEP.8: Hated themselves
- DEP.9: Felt like a bad person
- DEP.10: Felt lonely
- DEP.11: Felt unloved
- DEP.12: Felt not as good as others
- DEP.13: Felt they did everything wrong

Emotional Problems
- **EMO.1: Complained of sickness**
- **EMO.2: Many worries**
- EMO.3: Often unhappy
- EMO.4: Nervous in new situations
- **EMO.5: Many fears**

Hyperactivity
- HYP.1: Overactive
- HYP.2: Fidgeting
- **HYP.3: Easily distracted**
- **HYP.4: Does not think things out**
- **HYP.5: Bad attention**

Prosocial Scale
- PRO.1: Considerate of others
- PRO.2: Shared readily with others
- PRO.3: Helpful
- PRO.4: Kind to younger children
- **PRO.5: Volunteers to help**

Polygenic scores
- EA: PGS for educational attainment
- BMI: PGS for BMI
- DEP: PGS for depression
- ADHD: PGS for ADHD
- ANX: PGS for anxiety

© 2024 American Medical Association. All rights reserved.

References

1. Choi SW, Mak TSH, O'Reilly PF. Tutorial: a guide to performing polygenic risk score analyses. *Nat Protoc*. 2020;15(9):2759-2772. doi:10.1038/s41596-020-0353-1

2. Privé F. Polygenic scores and inference using LDpred2. Accessed November 24, 2022. https://privefl.github.io/bigsnpr/articles/LDpred2.html

3. Privé F, Albiñana C, Pasaniuc B, Vilhjálmsson BJ. Inferring disease architecture and predictive ability with LDpred2-auto. Published online October 12, 2022:2022.10.10.511629. doi:10.1101/2022.10.10.511629

4. Privé F, Arbel J, Vilhjálmsson BJ. LDpred2: better, faster, stronger. *Bioinformatics*. 2020;36(22-23):5424-5431. doi:10.1093/bioinformatics/btaa1029

5. giuliapiazza18. Example of analysis pipeline - Unweaving the polygenic web. Published online June 28, 2023. Accessed August 16, 2023. https://github.com/giuliapiazza18/Unweaving-the-polygenic-web-pipeline

6. Epskamp S, Cramer AOJ, Waldorp LJ, Schmittmann VD, Borsboom D. **qgraph** : Network Visualizations of Relationships in Psychometric Data. *J Stat Soft*. 2012;48(4). doi:10.18637/jss.v048.i04

© 2024 American Medical Association. All rights reserved.
