# Supplementary material for: Polygenic Scores and Networks of Psychopathology Symptoms
Source: JAMA Psychiatry. 2024 Jun 12;81(9):902–10. doi: 10.1001/jamapsychiatry.2024.1403 (PMC11170456; doi:10.1001/jamapsychiatry.2024.1403)
Supplement: Supplement 3. — Data sharing statement [file jamapsychiatry-e241403-s003.pdf]

Data Sharing Statement

Piazza. Polygenic Scores and Networks of Psychopathology Symptoms. *JAMA Psychiatry*. Published June 12, 2024. doi:10.1001/jamapsychiatry.2024.1403

Data
Data available: No

Additional Information
Explanation for why data not available: The ALSPAC resource is owned and provided by the University of Bristol. Data can be made available and accessed upon request, as detailed at https://www.bristol.ac.uk/alspac/researchers/access/. The TEDS resource is held by King's College London. Data can be made available, subject to a data sharing agreement, as detailed at https://www.teds.ac.uk/researchers/teds-data-access-policy.
